# Dose evaluation for skin and organ in hepatocellular carcinoma during angiographic procedure

**DOI:** 10.1186/1756-9966-32-81

**Published:** 2013-10-25

**Authors:** Daniela D’Alessio, Claudia Giliberti, Antonella Soriani, Livio Carpanese, Giuseppe Pizzi, Giulio Eugenio Vallati, Lidia Strigari

**Affiliations:** 1 Laboratory of Medical Physics and Expert Systems, Regina Elena National Cancer Institute, Via E. Chianesi 53, Rome, 00144, Italy; 2 Dipartimento Installazioni di Produzione e Insediamenti Antropici, INAIL, Via Alessandria 220/E, Rome, 00198, Italy; 3 Department of Radiology and Diagnostic Imaging, Regina Elena National Cancer Institute, Via E. Chianesi 53, Rome, 00144, Italy

**Keywords:** Skin dose measurement, Gafchromic film dosimetry, MOSFET dosimetry, Interventional radiology, Liver embolization

## Abstract

**Purpose:**

The purpose of this study is to evaluate the radiation dose in patients undergoing liver angiographic procedure and verify the usefulness of different dose measurements to prevent deterministic effects. Gafchromic film, MicroMOSFET data and DIAMENTOR device of the X-ray system were used to characterize the examined interventional radiology (IR) procedure.

**Materials and methods:**

A liver embolization procedure, the SIRT (Selective Internal Radiation Therapy), was investigated. The exposure parameters from the DIAMENTOR as well as patient and geometrical data were registered. Entrance skin dose map obtained using Gafchromic film (ESD_GAF_) in a standard phantom as well as in 12 patients were used to calculate the maximum skin dose (MSD_GAF_). MicroMOSFETs were used to assess ESD in relevant points/areas. Moreover, the maximum value of five MicroMOSFETs array, due to the extension of treated area and to the relative distance of 2–3 cm of two adjacent MicroMOSFETs, was useful to predict the MSD without interfering with the clinical practice. PCXMC vers.1.5 was used to calculate effective dose (E) and equivalent dose (H).

**Results:**

The mean dose-area product (DAP_DIAMENTOR_) for SIRT procedures was 166 Gycm^2^, although a wide range was observed. The mean MSD_GAF_ for SIRT procedures was 1090 mGy, although a wide range was experienced. A correlation was found between the MSD_GAF_ measured on a patient and the DAP_DIAMENTOR_ value for liver embolizations. MOSFET and Gafchromic data were in agreement within 5% in homogeneous area and within 20% in high dose gradient regions. The mean equivalent dose in critical organs was 89.8 mSv for kidneys, 22.9 mSv for pancreas, 20.2 mSv for small intestine and 21.0 mSv for spleen. Whereas the mean E was 3.7 mSv (range: 0.5-13.7).

**Conclusions:**

Gafchromic films result useful to study patient exposure and determine localization and amplitude of high dose skin areas to better predict the skin injuries. Then, DAP_DIAMENTOR_ or MOSFET data could offer real-time methods, as on-line dose alert, to avoid any side effects during liver embolization with prolonged duration.

## Introduction

The radiation dose to patients during complex Interventional Radiological (IR) procedures needs to be properly assessed. Prolonged fluoroscopy and a large number of image acquisitions can increase the Maximum Skin Dose (MSD) over the dose threshold for tissue reactions. This could increase the risk of skin injuries to patients [1,2].

The National Institute of Health (USA) has also published an advisory on the risks associated with interventional fluoroscopy [3], giving the approximate threshold dose levels at which skin injuries of increasing severity may occur, based on evidence accumulated in fluoroscopically guided interventions.

Moreover, when radiological interventions are prolonged for clinical reasons there is also an increase of the dose to the operators. Thus, dosimetric assessment is mandatory for optimizing radiation protection for both patients and operators, together with an accurate registration of technical data used for imaging procedures.

Measurements of Dose Area-Product (DAP) and of Entrance Skin Dose (ESD) could guide the physician when optimizing IR procedures, thereby reducing the delivered dose as much as possible, according to the clinical goal.

In order to measure the skin dose, some commercial devices for *in vivo* dosimetry, such as thermoluminescent dosimeters, radiochromic films, metal-oxide-semiconductor field effect transistors (MOSFETs) and ionization chambers, have been proposed and tested for IR procedures [4-9] or calibrated in phantom [10]. However, some guarantee real time information, while other a dose distribution over all the skin irradiated. In this report, a combination of both devices has been investigated.

In particular, the paper aims to compare the dosimetric results of liver angiographic procedures using Gafchromic films and MOSFET dosimeters, as well as to estimate organ and effective doses in medical X-ray examinations, radiography and fluoroscopy using a software program based on the Monte-Carlo method [11].

A quality control test for measurements with Gafchromic films (including scanner verification) and MOSFETs was performed, in order to assess the feasibility and reproducibility of routine dose verification.

## Materials and methods

### Tested procedure

SIRT is an angiographic procedure used in patients with hepatocellular carcinoma (HCC) or metastases.

The intra-hepatic brachytherapy, known by the acronym **SIRT** (Selective Internal Radiation Therapy), is an oncologic therapy based on selective regional intra-arterial administration of resin microspheres that are loaded with Yttrium-90, (^90^Y) radioisotope (SIR-spheres® Sirtex Medical Europe). A SIRT therapy is normally performed with two procedural steps. A few days before the injection of Yttrium spheres an embolization of the gastroduodenal or other arteries is performed to avoid inadvertent delivery of Y-microspheres combined with a shunt scintigraphy to exclude significant shunting into the lung using ^99m^Tc-MAA (MacroAggregates of Albumin).

In particular in this paper the second phase of the entire SIRT procedure was evaluated, that represents the therapeutic phase. In this phase the dose to tumour and normal tissue is due to the β-particles of ^90^Y (which is a pure β-emitter with a half-life of 64.1 hours), while the irradiation of healthy tissues from both bremsstrahlung originated from β-particles of ^90^Y and photons emitted from X-ray tube.

Microspheres loaded with the radioisotope ^90^Y have a diameter of about 32 μm, a size sufficiently small to allow the delivery through the vascular system of the liver by super selective catheterization of the feeding tumour arteries, thus reaching the peripheral vessels. Microspheres are trapped in the tumour vascular bed delivering a high dose of radiation locally with an average penetration depth of 2.5 mm. The effect of ^90^Y lasts about 14 days. Its activity decays almost completely during the first two weeks after treatment and the irradiated cells undergo necrosis. More details can be found in another paper [12].

The duration of SIRT procedures depends on the complexity of the arterial anatomy and number of vessels involved [13]. These procedures are minimally invasive, routinely performed in the Interventional Radiology Department of our Institute for therapeutic aim and generally require an exposure time of several minutes.

### Patient selection

The total number of enrolled patients were 12 (8 male and 4 female) underwent SIRT procedure.

The median age was 63.0 years (range: 35–79), the median height was 167 cm (range: 155–182), the median weight was 69.9 kg (range: 43–90) and the median BMI was 25.0 kg/mq (range: 17.9-32.3).

### Angiography device

We used as digital angiography device a C-arm Innova 4100 of General Electric (GE Healthcare, Buc, France), which has an X-ray tube under the couch and a digital flat panel with iodure of cesium (CsI) with a high sensitivity and efficiency at X-ray. The tube Performix 160 A has an 11° anode angle, accelerating potential from 50 to 125 kV, current from 1 to 400 mA, focal spot size of 0.3-0.6-1 mm; the digital flat panel (with a pixel size of 0.2 mm) has a maximum field of view of 40 cm diameter, with three fields (with 32, 20 and 16 cm diameter) with different zoom factors. The angiography device works for this procedure in normal modality for cine and in low modality for fluoroscopy.

A quality control (QC) program of the X-ray unit was regularly performed, including measurements of the radiation output, half-value layer (HVL), peak kilovoltage (kVp).

### Dosimetric devices and software

To assess the dose delivered to the skin, the following dosimetric devices were used: Gafchromic films type XR-RV2 manufactured by ISP (International Specialty Products, Wayne NJ) and MicroMOSFET dosimeters type TN-1002 RDM (Best Medical, Canada, Ltd.).

In particular, XR-RV2 were used to measure the surface dose distribution map and to evaluate the MSD, while the MicroMOSFETs were used to assess the dose value in specific points of the investigated surface.

The exposure values (air kerma) were measured using the multimeter PMX III (RTI Electronics AB, Mölndal, Sweden), calibrated at a Secondary Standard Dosimetry Laboratory in Sweden.

The DAP values were obtained using the DIAMENTOR M4 KDK (PTW, Freiburg, Germany), i.e. an on-board dosimetry device of the C-arm Innova 4100.

The DAP meter readings were in agreement with PMX data in the range from 0 to 400 Gycm^2^ with reproducibility within ± 30%. Calibration of multimeter PMX III was done by ENEA (Italy) every year, in collaboration with medical physicists of the Laboratory of Medical Physics and Expert Systems of our Institute.

To measure the radiographic ESD, we refer to the report of AAPM Task Group 8 for C-arm fluoroscopy devices [14].

Estimated doses to patient organs were calculated with the software PCXMC developed by STUK (Radiation and Nuclear Safety Authority, Helsinki, Finland).

### Experimental setup for calibration

**Figure 1:**
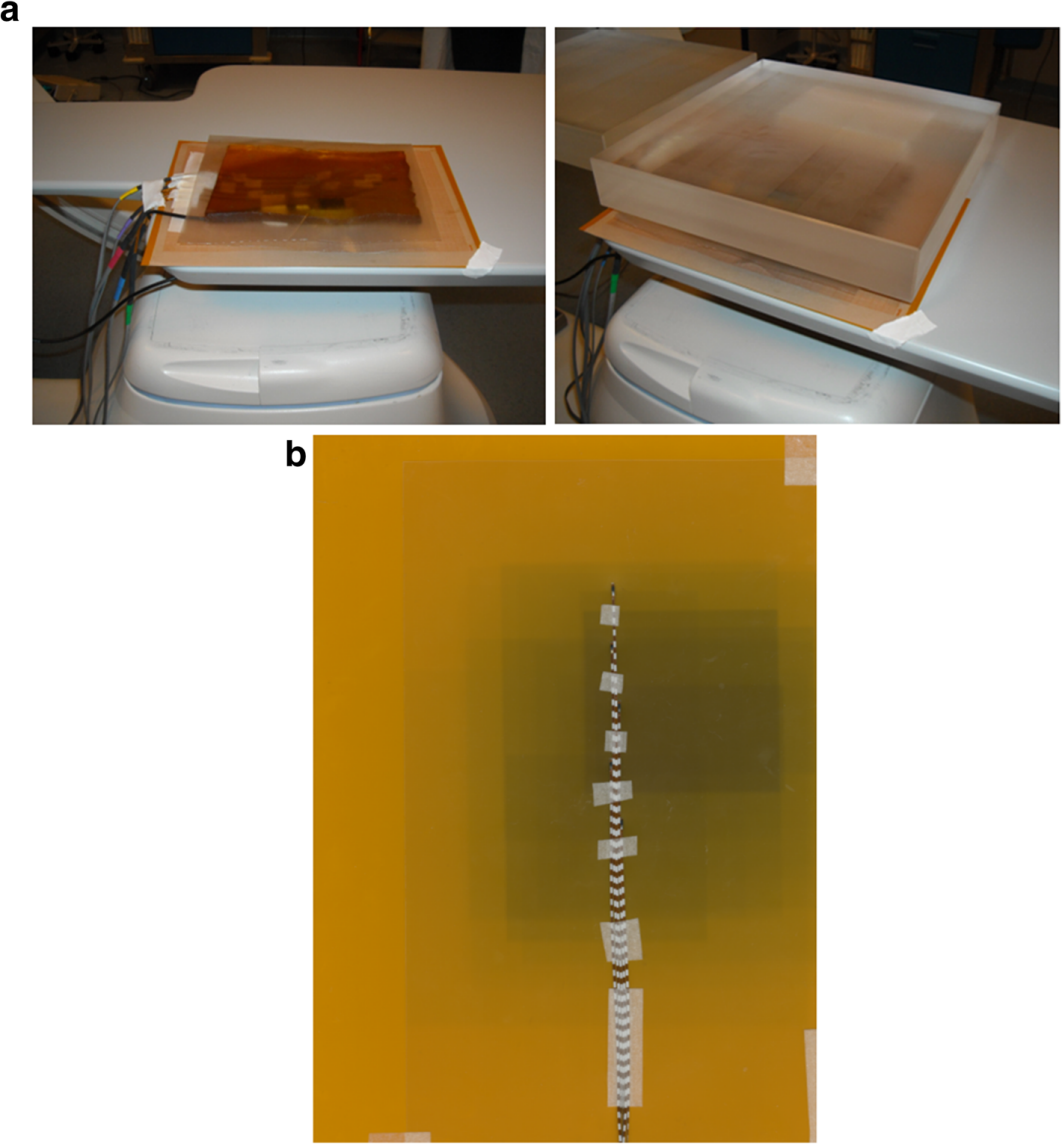
a) Experimental setup for gafchromic film and MicroMOSFET calibration; b) MicroMOSFETs array on the gafchromic film.

The Gafchromic films, MicroMOSFETs and PMX were placed between couch and patient-like phantom [15] to obtain the ESD values (Figure 1a) using the experimental set-up described in the following. Dosimeters were placed on the couch in the centre of FOV (Field Of View) in order to be included within the minimum used FOV (i.e. 16×16 cm^2^) where the beam was assumed to be uniform. The patient-like phantom was of PMMA with thickness 20 cm and size 30 × 30 cm^2^[16].

In particular the irradiation was performed in service-mode using 90 kV, such as that used in clinical practice, with step of 25 mAs in order to obtain doses from 4 to 24 mGy.

All parameters (kV, mA, dose) were measured and registered with PMX placed as shown in Figure 1a.

The reported DAP was corrected for the factors of calibration of the chamber but not for the attenuation of the couch.

An anti-scatter grid was used to protect the digital detector from the scattered radiation.

This setup allows us to perform a relative calibration of gafchromic and MOSFETs using the PMX at a reference depth (couch thickness) including the backscatter radiation (due to patient-like PMMA); similar to the approach “in-phantom method” reported in the AAPM 61 [17].

### Gafchromic films XR-RV2

The XR-RV2 are specific for the IR procedures guided by fluoroscopy. Each sheet has a size of 14″×17″ and is sensitive to wide dose ranges 0.1 Gy to 50 Gy [18].

From a sheet of Gafchromic film, pieces about 4.0×3.0 cm^2^ were cut and signed, taking into consideration the dimension of the sheet. A piece of unexposed Gafchromic was used as unexposed reference in order to assess the background.

The images were acquired 24 hours post irradiation using the EPSON Expression 10000XL flatbed colour scanner in reflection mode.

The program Picodose X 8.0 PRO software (Tecnologie Avanzate, Torino, Italy) was used to calibrate the X-ray exposed films.

The dose response of Gafchromic film in different colour components, red-green-blue (RGB), shows that the sensitivity is maximized when the red channel component was used, according to Alva et al. [19].

As QC procedure, post-exposure values were evaluated 24 hours post-irradiation and then at different times (every 7 days) for a period of 35 days, showing a variation lower than about 3% at low doses and 1% at doses higher than 10 Gy (data not shown).

### MOSFETs

To determine the dose in points of interest (POIs), such as the area where the MSD is expected, MicroMOSFETs dosimeters (Metal Oxide Silicon Field Effect Transistors) TN-1002RDM model, particularly indicated for the *in vivo* dosimetry [20], were chosen for their feature to give an on-line read-out, immediately providing the dose measurement.

The small size of detectors (active area 0.1 mm^2^) makes them particularly suitable for point dose measurements. MOSFET and MicroMOSFET have the same active detection area but different package: only 1.0 mm thickness of bulb, 0.3 mm thickness and 1.0 mm width of flex cable for MicroMOSFET.

Unfortunately a single MicroMOSFET cannot be useful to determine the MSD, because the position of the maximum skin dose is unknown. Thus an array of five MicroMOSFETs with a spatial distribution over a region of interest has been used to investigate multiple points of irradiated area. The relative distance of two adjacent MOSFETs was 2–3 cm. In case of SIRT, the maximal dose is expected in an area close to the diaframma, thus the MOSFET positioning is quite fast and feasible.

MOSFET detectors were connected through a bias supply TN-RD-22 to the reader and were managed by a personal computer. An initialization set-up was necessary before the irradiation and the information was preserved until the next reading e/o initialization. Bias supply was set at high sensitivity mode. The usage of a X-ray beam of 80/90 kV required a complete characterization of the dosimetry system. In particular, dosimeters were irradiated using the same set-up already described for the Gafchromic films calibration (Figure 1a).

For each MicroMOSFET, an individual calibration factor F (mGy/mV) was determined, as mean value of 3 irradiation readings, each obtained by a dose ranging from 200 to 4000 mGy. Doses of irradiation were measured by the PMX positioned next to the MOSFET detectors.

To check position of MOSFETs on the Gafchromic film a signed transparent paper was used as shown in Figure 1b. Moreover, the position of the MOSFET can be checked in the angiographic image during the SIRT procedure without interfering with the clinical examination.

### PCXMC

The program based on Monte-Carlo method, called PCXMC, vers. 1.5, (STUK - Radiation and Nuclear Safety Authority, Helsinki, Finland) was used to estimate organ and effective doses in medical X-ray examinations, radiography and fluoroscopy [11]. This program allows to simulate the physical interaction by specifying the parameters relating to the examination and, consequently, to calculate the dose to the organs for patients of different ages and sizes by using a phantom (Cristy) in which the morphology of different organs is reproduced. In addition to the patient data (age, height and weight), those of the radiation beam (focus-detector distance, size of the radiation field, beam projections) are also required, and the values of the tube voltage, the anode angle, the tube filtration which can be specified by two different filters of appropriate materials and the DAP.

The value of the DAP (mGycm^2^), as the input dose quantity, was used.

The effective dose was calculated using the PCXMC based on equivalent dose and weight factors from ICRP 103 [21] in order to assess the risk of the stochastic biological effects.

The localization of the FOV with respect to the patient geometry was determined in order to identify the organs irradiated during the IR procedures.

### Film positioning during patient study

The Gafchromic films and MOSFETs were placed close to each other on the table-couch under the patient (between couch and patient). They were centred by an IR physician in the area of the patient where more irradiation is expected. The film size was 30x40 cm^2^, which would cover a large area of the dose distribution.

If the examination provided lateral projections, additional film was employed on the sides of the patient.

For each IR procedure, we registered in a report the following data; patient age, height and weight, FOV, SSD, projections (which are fixed at 0° in this clinical approach), kV, mA, ms, mm of Cu, type of acquisition (i.e. graphy and scopy), exposure time (total X-ray time), and DAP provided by the DIAMENTOR.

### Data analysis

The DAP and ESD values were compared with ESD based on Gafchromic films and MOSFETs, both placed in the area where the MSD is expected. Effective dose (E) and equivalent dose (H), including the skin equivalent dose, were also calculated using PCXMC. All data collected during the patient examination were correlated to each other as well as with exposure conditions, patient characteristics and treatment technique. All data were compared using the Person correlation coefficients. A p-value lower than 0.05 was assumed to be statistically significant. The data analysis was performed with an R-package ver. 14 [22].

## Results

### Calibration curves

**Figure 2:**
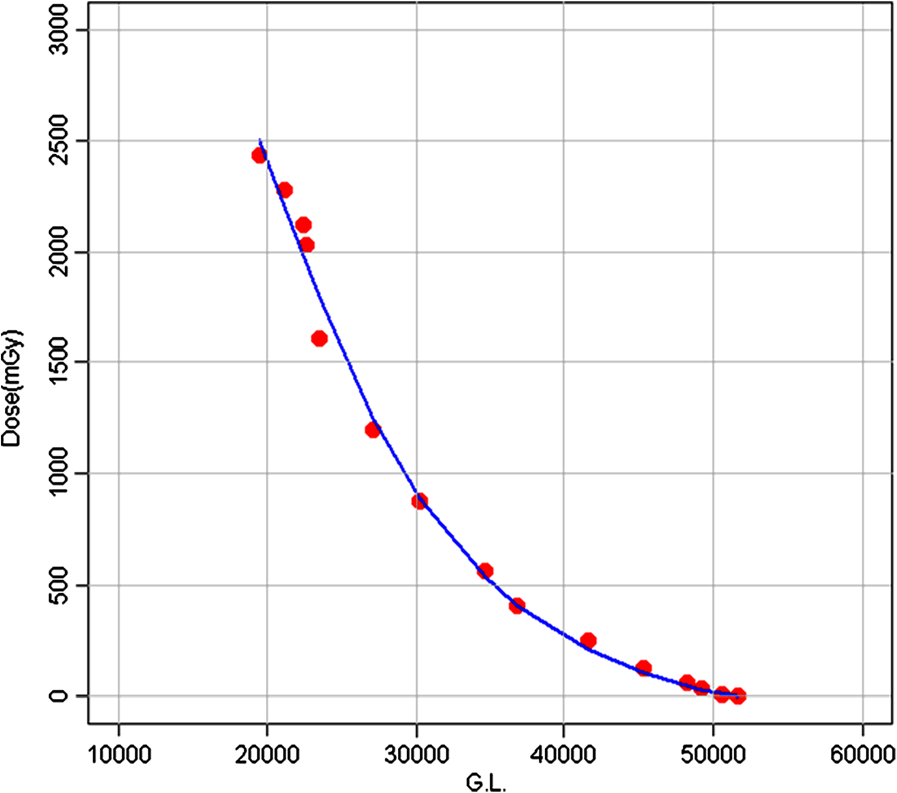
Calibration curve of the gafchromic films XR-RV2 (i.e. Dose (mGy) versus Gray Levels).

The calibration curve obtained with Gafchromic XR-RV2 films, using the red channel component, is shown in Figure 2. The calibration curve is a polynomial function of degree 3 (Dose(mGy) = (1.0144^3) - (5.9082^-2)GL + (1.1950^-6)GL^2 - (8.3602^-12)GL^3). The uncertainty is within 3%, which is generally accepted for this type of device.

The ESD by MOSFETs was given by M · F, where M is the reading (mV) of MOSFETs positioned under the patient, on the skin of the back. Five MOSFETs were used during the measurements with individual F values of 0.184 and 0.193 mGy/mV. Each MOSFET showed a linear response with dose within 2.5% at the calibration radiological energy.

The overall uncertainty of the MOSFET response, which includes the uncertainty of the calibration factor (3% 1SD), the angular dependence (2% 1SD), linearity with dose (2% 1SD) and digital angiography device output fluctuation (1% 1SD) was within ±4% (1SD).

### Data of liver IR procedures

During fluoroscopy and image acquisition, the tube potential was controlled by automatic exposure control (AEC) and varied from 80 to 95 kV depending upon the thickness of the patient, selection of zoom and SSD. The mean time duration of fluoroscopic for patients who underwent SIRT was 18 min, while the mean number of frames acquired during fluoroscopy SIRT was 2166. The main parameters are reported in Table 1.

**Table 1:** Values (mean and range) of the main parameters of irradiation

***Parameters***	***Mean number of frames***	***Mean X-ray time (min)***	***Mean duration fluoroscopy time (min)***	***Mean DAP***_***DIAMENTOR ***_ ***(Gycm***^***2***^ ***)***	***Mean ESD***_***DIAMENTOR ***_ ***(mGy)***	***Mean MSD***_***GAF ***_ ***(mGy)***
***SIRT***	2166	18 (8-26)	16 (6-22)	166 (25-400)	1050 (410-2290)	1090 (540-1880)

### Dosimetric results

DAP up to 400 Gycm^2^ were measured. In particular, the mean DAP was 166 Gycm^2^ but a wide range, from 25 to 400 Gycm^2^, was observed.

There was a poor correlation between DAP and fluoroscopy time, as well as between DAP and number of frames; while there was acceptable correlation between DAP and number of frames registered within the study involving each patient thanks to the FOV amplitude related to the lesion dimension. The FOV mainly used was 32×32 cm^2^.

**Figure 3:**
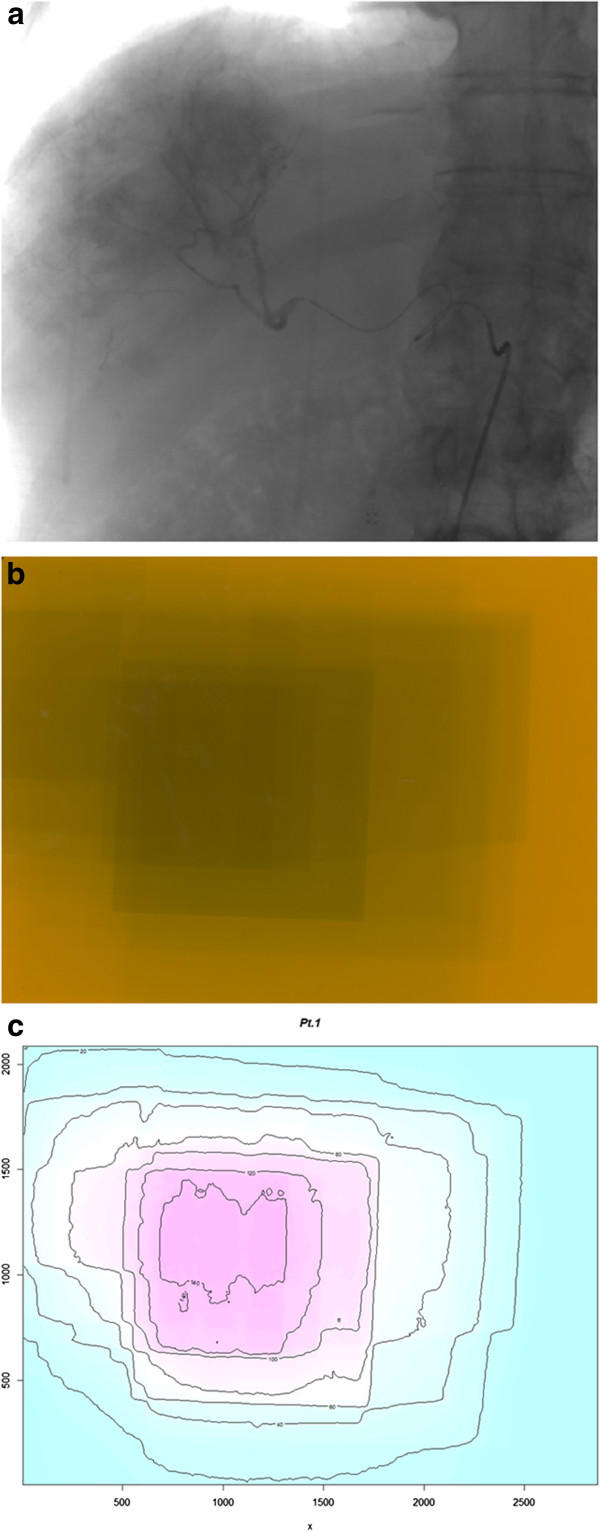
a) Angiographic image, b) acquired gafchromic film and c) isodose levels on the dose distribution map of a representative patient.

Furthermore, a large variability was also observed in the dose distribution map. The angiographic image, the acquired Gafchromic and the isodoses on the dose distribution map of a representative patient are shown in Figures 3a, 3b and 3c. Both the MSDs as well as the extension of the region of high doses (over 1400 mGy) or lower dose (less than 400 mGy) showed a wide variability.

**Figure 4:**
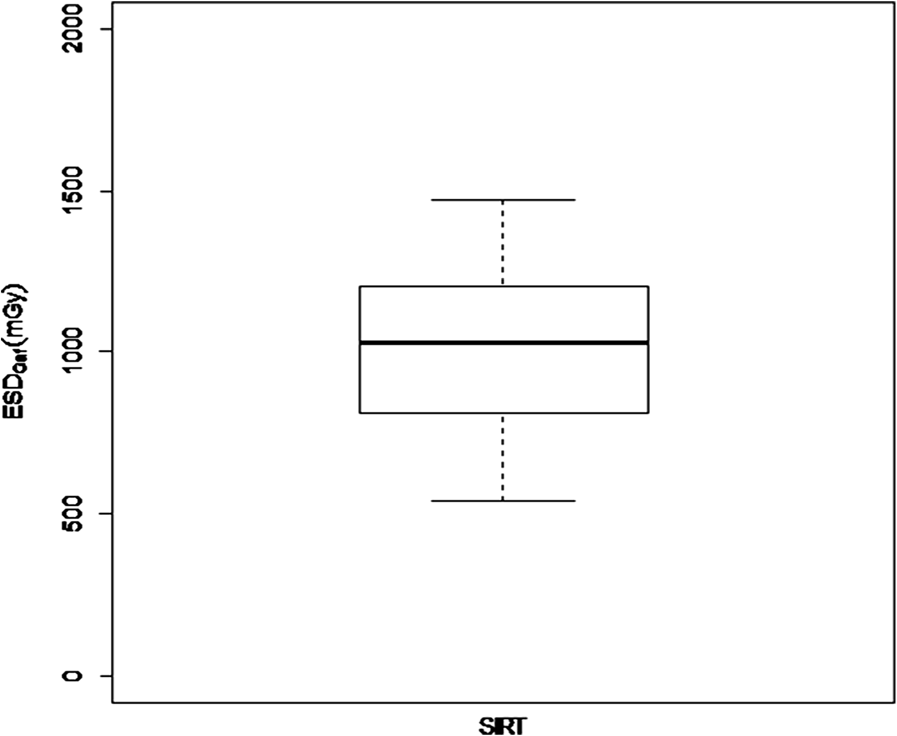
Box plot of ESD by Gafchromic indicating median and range of values.

The MSD, for each dose distribution map, was somewhat variable in our cohort with a mean value of 1090 mGy and a range from 540 to 1880 mGy (Figure 4).

**Figure 5:**
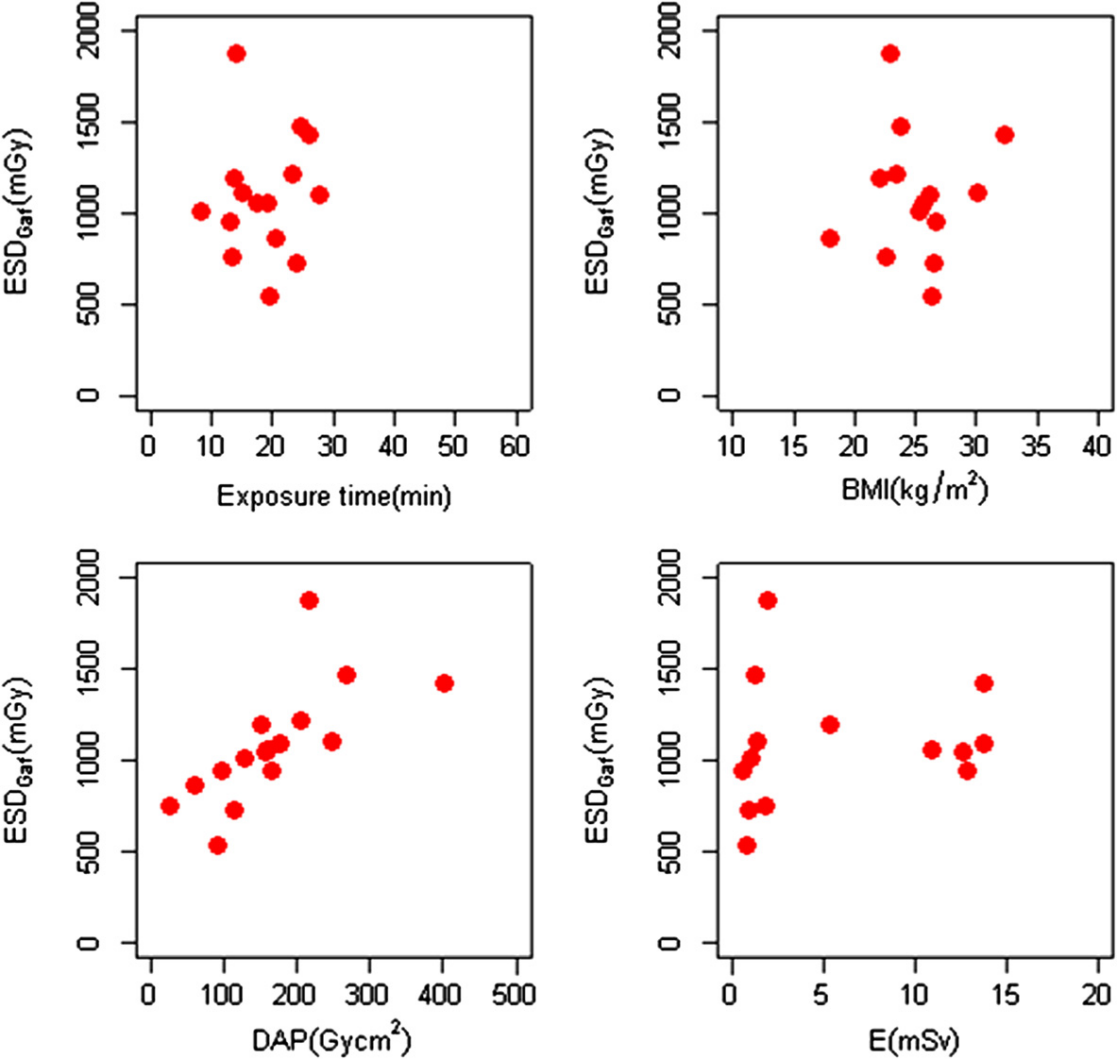
ESD by Gafchromic vs a) exposure time, b) BMI, c) DAP and d) effective dose.

The MSD versus exposure time, BMI, DAP and effective dose E is reported in Figure 5. The MSD by Gafchromic and DAP showed a statistically significant positive correlation (p = 0.002), with a Pearson correlation coefficient of 0.71. The remaining variables did not seem statistically significant when correlated with the MSDs by Gafchromic.

Moreover, comparing the ESD provided by DIAMENTOR and those determined as MSD by Gafchromics, we observed differences were lower than 20% in 8/12 (67%) patients and over 50% in 3/12 (25%) patients. The above differences could be due to the different contribution of back scattered radiation incident on the DIAMENTOR and on Gafchromics. This probably because DIAMENTOR provides a cumulative value not representative of the MSD, which depends on the beam focusing as well as to the change of FOV position (both variable during procedure).

**Figure 6:**
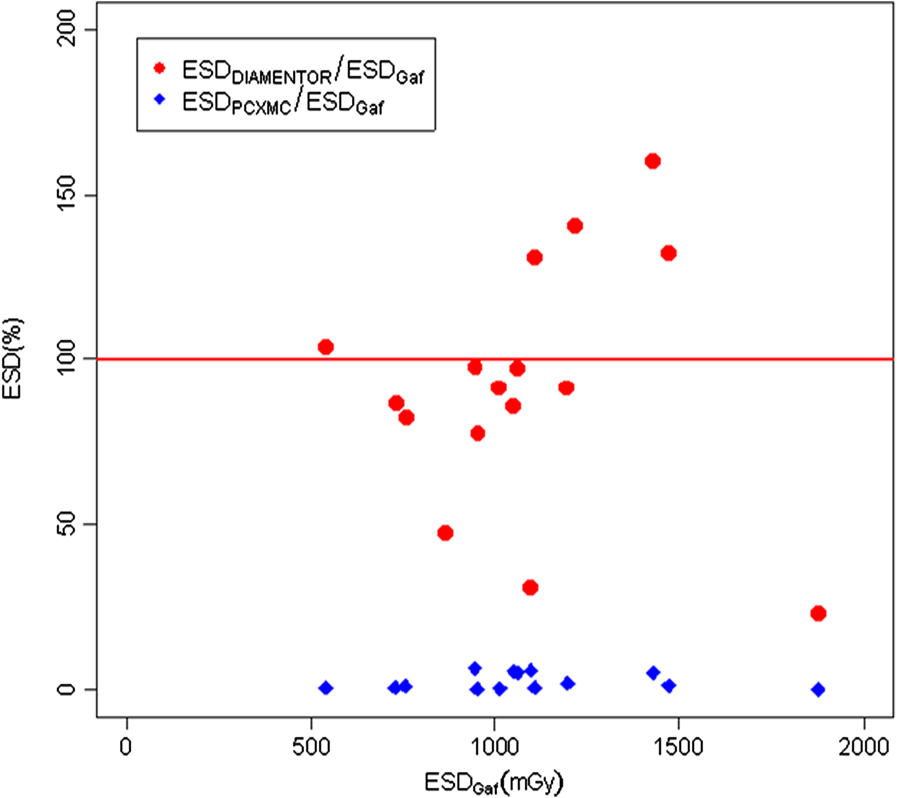
ESD from DIAMENTOR (red circle) or PCXMC (blue diamond) against ESD assessed by Gafchromic.

As expected, the ratio between average skin dose provided by PCXMC and by the maximum ESD measured with Gafchromic versus the ESD by Gafchromic was lower than one in all patients (Figure 6). This is probably due to the fact that the other variables are representative of the median values of ESD and not of the maximum registered on Gafchromic film.

Before the beginning of the IR procedure the array of MicroMOSFETs was placed from Radiologists at the centre of FOV.

The maximum value of five MicroMOSFETs array, due to the extension of treated area (at least about 10×10 cm^2^) and to the relative distance of 2–3 cm of two adjacent MicroMOSFETs, was useful to predict the MSD without interfering with the clinical images. In fact the impact on the clinical images was judged trivial from all IR radiologists, since the size of this detector are comparable with those of microcatheters used in this type of IR procedure.

MicroMOSFETs and Gafchromic data were in agreement within 5% in homogeneous area (mainly where the MSD were registered) and within 20% in high dose gradient regions.

The MicroMOSFETs array approach makes the ESD evaluation possible during the liver angiographic procedure. The analysis of the data collected, with reference to the type and the timing of the procedure, could allow the identification of optimization criteria. For example, it is possible to perform the reading of dosimeters in a certain instant and estimate if it is exceeded a certain value of skin dose.

**Figure 7:**
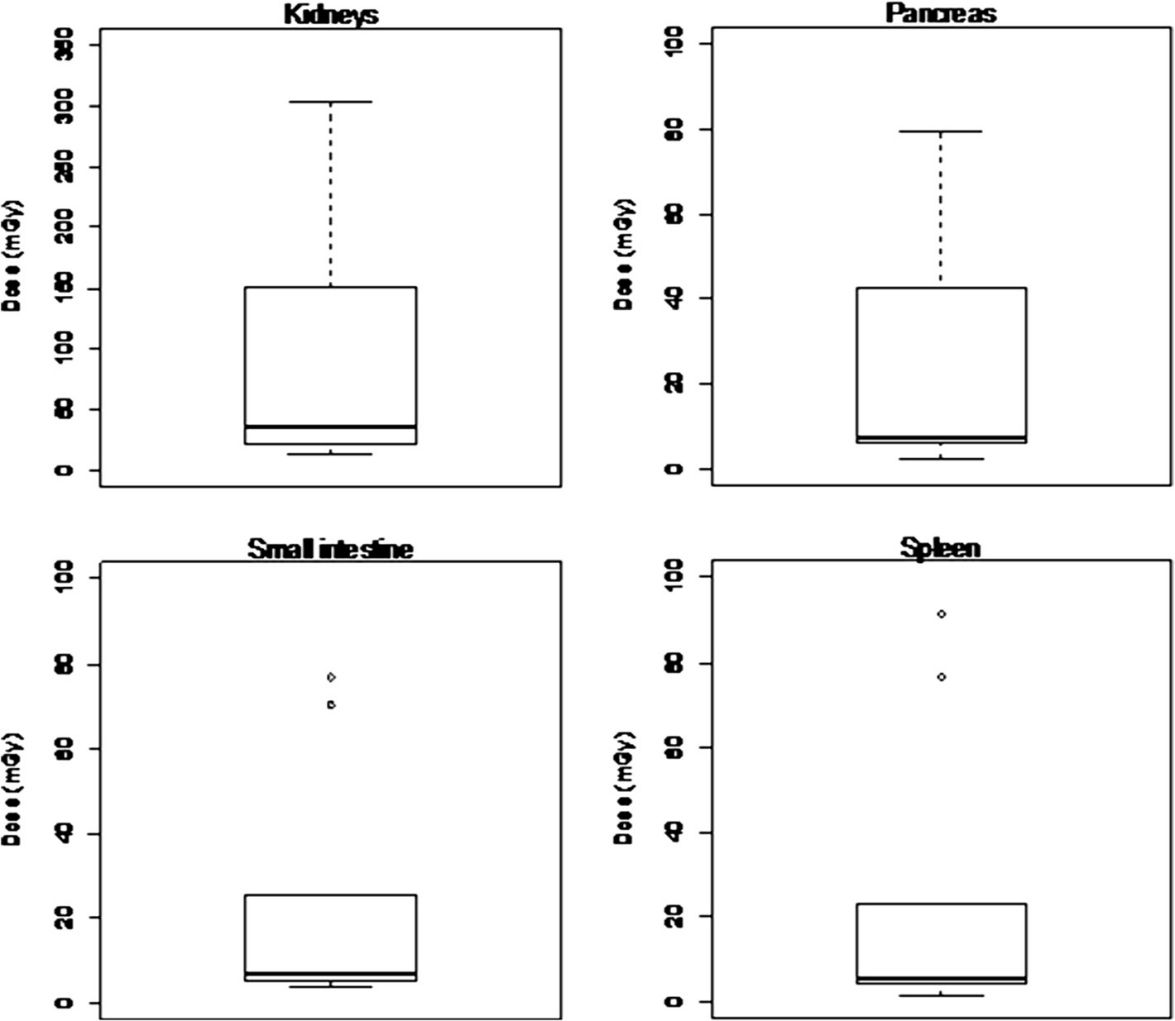
Dose to organs calculated by PCXMC a) kidney, b) pancreas, c) small intestine and d) spleen.

Finally, doses to organs receiving the highest doses, as calculated by PCXMC, in our cohort of patients were shown in Figure 7. The values that were outside the ranges were plotted as a circle. In particular, the mean H in critical organs as calculated by PCXMC was 89.8 mSv for kidneys, 22.9 mSv for pancreas, 20.2 mSv for small intestine and 21.0 mSv for spleen. While the mean E was 3.7 mSv (range: 0.5-13.7).

## Discussion

While for most of the diagnostic procedures, the dose threshold of 2–5 Gy for deterministic effects for skin is not reached, this situation can be much different for embolizations [13,23,24].

In fact, the MSD strongly increases with the time of fluoroscopy, the DAP, as well as with the number of acquired images.

At present the major part of interventional radiology equipments do not show an estimation of MSD in the clinical practice, because only an automatic exposure control is currently integrated with the C-arm machine.

Moreover, the use of MicroMOSFETs array can be useful to assess the ESD in some significant points; while Gafchromic films can be used to measure a distribution map of ESD. In particular, the MOSFET for their feature to give an on-line readout, immediately providing the dose measurement. Being the MicroMOSFETs and the MSD by Gafchromic in agreement within 5% in homogeneous area, the use of MicroMOSFETs make possible an on-line alert level to permit physician a possible consequent adjustment during the procedure, or thereafter the use of some biological response modifiers which has been suggested to potentially help reduce late reactions in many tissues [2].

In this work we employed both devices on a standard phantom as well as in 12 patients during IR procedure for liver embolizations in order to estimate the MSD.

For liver embolization, large differences in clinical protocol and technical parameters of the X-ray system, explaining the measured dose variations were observed in our cohort. In particular, the mean exposure time for SIRT was 18 min (range: 8–26 min), while the mean fluoroscopy time was 16 min (ranging from 6 to 22 min). Our fluoroscopy time is larger than the value reported by Bor et al. [25], indicating 7.7 min (range: 1.8-13) for therapeutic hepatic procedures. However, these values varied according to the complexity of the angiographic procedure, but also on the patient anatomy.

The measured DAP in our cohort was similar to that reported by Livingstone et al. [13] (i.e. 142 Gycm^2^, range: 44–292), McParland [26] (i.e. 136 Gycm^2^, range: 18–267). The high DAP values registered during hepatic embolization were probably due to the use of a large field area of irradiation without precise collimation in some phases.

Larger MSD values than those reported in our cohort have been reported by other authors [13] ranging from 130 to 2990 mGy during abdominal embolizations; in some cases overcoming the threshold of deterministic effects for early transient erythema (2–5 Gy) or approaching temporary epilation (5–10 Gy) [23,24].

Our results support the need for an accurate dose determination during liver embolizations when prolonged procedures are to be performed. On the one hand, the complexity of the pathology for IR procedures could significantly increase MSD.

There was a good correlation between DAP and number of frames (obtained for each patient); while there was a poor correlation between DAP and fluoroscopy time, as well as between DAP and number of frames, according to Ref. [27]. A correlation was also found between MSD and DAP value for liver embolizations.

This correlation makes it possible to estimate the MSD from the DAP values and/or determine a trigger level using appropriate coefficient determined by the calibration of MOSFETs, in order to better estimate the MSD in IR procedures.

Critical organs for SIRT were kidneys, pancreas, small intestine and spleen, according to literature [28]; while the liver received a low mean dose due to the large liver volume (compared to the lesion) and to use of FOV collimation. Moreover, in SIRT procedures the liver receives a dose from bremsstrahlung during the therapeutic phase, as well as an additional dose in the diagnostic/pre-therapeutic phase due to ^99m^Tc-MAA and fluoroscopy study. In this paper we focused the investigation on interventional phase because the exposure time is generally longer with the possibility to overcome the skin tolerance dose.

The mean E was 3.7 mSv (range: 0.5-13.7) for SIRT was lower than the value reported for generic abdominal angiography (i.e. 8 mSv) [29,30], probably due to the variability of the IR procedure involving the abdominal region.

## Conclusion

Our data indicate the possibility to overcome the deterministic effects during the liver embolizations when a prolonged duration is needed for the complexity of the IR procedure or patient anatomy. DAP or MOSFET data could represent possible real-time methods to avoid side effect, with the first offering the advantage of being integrated with the C-arm device and the latter as an independent instrument. In particular the maximum value of MicroMOSFETs array can be used as on-line dose alert. This is particularly useful in case of variations of a technique already established or implementation of a new procedure.

ESD maps obtained from Gafchromic films could be useful to retrospectively study the exposure characteristics of an IR procedure and plan patient exposure optimization by determining localization and amplitude of high dose skin areas.

A good correlation was found between the DAP value and the ESD measured with Gafchromic for each patient of our cohort. However, ESD showed the full planar distribution and DAP only a fully comprehensive value that might over or under estimate the real MSD. In addition an ESD map could be used as a proven method to report the effective MSD and is acceptable as regards precision and accuracy for clinical measurements of skin dose.

In conclusion, the aim of this study is to evaluate the radiation dose in patients undergoing liver angiographic procedure and verify the usefulness of different dose measurements to prevent deterministic effects as well as to validate a method for in vivo dosimetry with different devices, which is particularly useful in case of implementation of a new procedure in the clinical practice.

## Competing interests

The authors declare that they have no competing interest.

## Authors’ contributions

DD and LS designed the study and collected data and measurements. LC, GP and GEV delivered treatments and provided clinical data. DD, CG, AS and LS carried out data interpretation. DD and LS drafted the manuscript. All authors read and approved the final manuscript.
